# Artificial Intelligence and Circadian Thresholds for Stress Detection in Dairy Cattle

**DOI:** 10.3390/s25216544

**Published:** 2025-10-24

**Authors:** Samuel Lascano Rivera, Luis Rivera, Hernán Benavides, Yasmany Fernández

**Affiliations:** 1 Research Department, State Polytechnic University of Carchi, Tulcán 040101, Ecuador; hernan.benavides@upec.edu.ec (H.B.); yfernandezf@upec.edu.ec (Y.F.); 2 Postgraduate Program, Faculty of Systems and Informatics Engineering, National University of San Marcos, Lima 15081, Peru; rivera@uenf.br; 3 Mathematical Sciences Laboratory, State University of Norte Fluminense, Rio de Janeiro 28013-602, Brazil

**Keywords:** bovine stress, machine learning, circadian rhythm, activity level, Fast Fourier Transform, LSTM

## Abstract

This study investigates stress detection in dairy cattle by integrating circadian rhythm analysis and deep learning. Behavioral biomarkers, including feeding, resting, and rumination, were continuously monitored using Nedap CowControl sensors over a 12-month period to capture seasonal variability. Circadian features were extracted using the Fast Fourier Transform (FFT), and deviations from expected 24 h patterns were quantified using Euclidean distance. These features were used to train a Long Short-Term Memory (LSTM) neural network to classify stress into three levels: normal, mild, and high. Expert veterinary observations of anomalous behaviors and environmental records were used to validate stress labeling. We continuously monitored 10 lactating Holstein cows for 365 days, yielding 87,600 raw hours and 3650 cow-days (one day per cow as the analytical unit). The Short-Time Fourier Transform (STFT, 36 h window, 1 h step) was used solely to derive daily circadian characteristics (amplitude, phase, coherence); STFT windows are not statistical samples. A 60 min window prior to stress onset was incorporated to anticipate stress conditions triggered by management practices and environmental stressors, such as vaccination, animal handling, and cold stress. The proposed LSTM model achieved an accuracy of 82.3% and an AUC of 0.847, outperforming a benchmark logistic regression model (65% accuracy). This predictive capability, with a one-hour lead time, provides a critical window for preventive interventions and represents a practical tool for precision livestock farming and animal welfare monitoring.

## 1. Introduction

Stress in cattle represents a critical challenge for modern livestock farming due to its direct impact on animal welfare and productivity. Factors such as intensive management practices, adverse environmental conditions, and disruptions to daily routines trigger physiological and behavioral responses that reduce feed intake, alter resting patterns, and increase susceptibility to disease [1,2]. These alterations adversely affect milk quality and yield, reproductive efficiency, and overall profitability of the sector [3,4]. Consequently, the early and accurate detection of stress is essential for implementing management measures that promote animal welfare and productive sustainability.

Precision livestock farming has integrated smart sensors capable of recording real-time indicators such as rumination, feed intake, and resting time [5,6]. These devices enable identification of subtle variations in animal behavior; however, they present limitations in differentiating specific states of health, reproduction, or stress [7,8]. In this context, the analysis of circadian rhythms emerges as a promising approach. These biological rhythms regulate daily activity, and their disruption can reflect conditions of stress or illness [9,10]. The FFT [11] facilitates the decomposition of activity patterns into their frequency components, allowing for the detection of alterations in 24 h circadian cycles and their correlation with physiological states. This technique has shown potential for identifying variations that traditional methods fail to capture [12]. Concurrently, advances in artificial intelligence (AI), particularly Long Short-Term Memory (LSTM) neural networks, have revolutionized the analysis of time-series data by capturing long-term dependencies and robustly handling noisy data [12,13]. These characteristics make LSTM ideal tools for modeling complex circadian dynamics and detecting alterations associated with stress.

Table 1 summarizes the most relevant prior research lines: (i) prediction of thermal stress using classical or deep machine learning, which is typically reactive and lacks explicit circadian modeling [3,14,15]; (ii) circadian analysis of activity, offering descriptive contributions but lacking real-time predictive components [12]; and (iii) behavior classification from accelerometry data (ML/DL) that is not specifically focused on stress or does not integrate a formal circadian profile [16]. Collectively, these studies demonstrate significant progress (see Table 1); however, they fail to fully integrate a circadian profile extracted via FFT with a sequence model (LSTM) that, given the last 24 h circadian reconstruction, predicts the stress level one hour in advance (here, the sequence model refers to a Long Short-Term Memory (LSTM) network that processes time-ordered inputs; the FFT serves as a deterministic preprocessing step to extract circadian components and does not constitute a second classifier).

This study proposed a novel approach that combines FFT to extract a 24 h circadian profile with an LSTM model to classify and predict three stress levels (normal, mild, high) with a one-hour anticipation window. The model was validated on 12 months of longitudinal data from Nedap CowControl sensors. It achieved an accuracy greater than 80% and a competitive macro One-versus-Rest (OvR) AUC score. This contributed to a predictive and proactive system that overcomes the reactive limitations and the absence of circadian integration identified in Appendix A.

## 2. Materials and Methods

The study was conducted at the San Francisco experimental center of the State Polytechnic University of Carchi (Ecuador), located at 2950 m above sea level, under semi-intensive stabling conditions. The sample consisted of 10 lactating Holstein cows, aged between 3 and 6 years, clinically healthy and maintained under homogeneous feeding and management conditions. Although the sample size was limited, this was compensated for by a 12-month longitudinal monitoring period (January–December 2024), which captured circadian and seasonal variations in activity patterns [3,17].

### 2.1. Sensors and Data Acquisition

**Figure 1:**
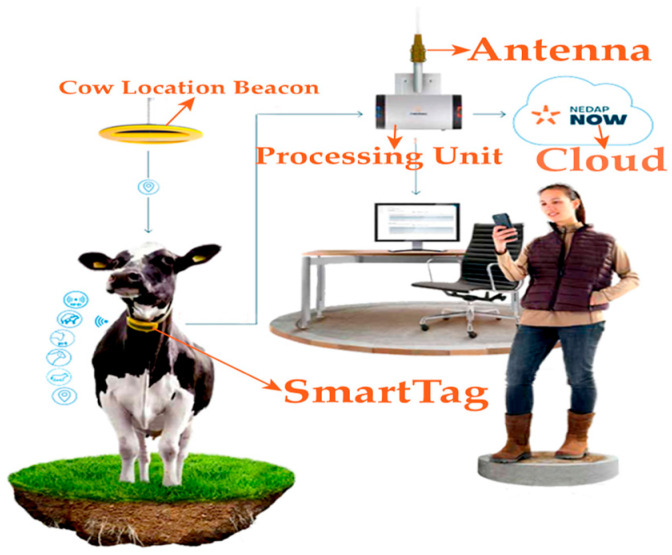
Nedap SmartTag collar architecture.

The Nedap CowControl^®^ system (Nedap Livestock Management, Groenlo, The Netherlands) was employed, utilizing SmartTag intelligent collars (codes FERP4, 3rd generation). Each SmartTag incorporates a triaxial accelerometer that transmits data at 434 MHz with a power of <1 mW e.r.p., features an IP67 protection rating, and has an operational temperature range from −10 °C to 50 °C. The battery-powered (3V DC) RFID transmitters continuously record feeding, rumination, resting, locomotion, and inactivity, storing data locally for 24 h before transmission to the CowControl antenna (outdoor range ≥ 100 m). Data were processed by specialized algorithms on the cloud-based platform to detect reproductive, health, and behavioral patterns, generating automated alerts and analyses for both individual animals and the herd [14]. These data were used to derive day/night activity indices and 24- to 36 h time series for circadian analysis, as detailed in Section 2.3, Section 2.4 and Section 2.5 (Figure 1 and Table 1).

**Figure 2:**
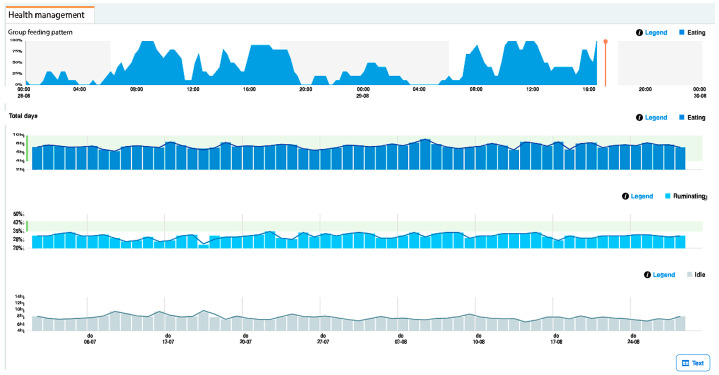
Example of a Nedap CowControl dashboard view showing a cow’s hourly feeding, resting, rumination, and other activities.

Although the cohort comprised 10 lactating Holstein cows, we recorded hourly time series for 12 months, generating 87,600 temporal measurements that constitute repeated observations nested within individual animals, not independent replicates. The experimental unit was the cow (n = 10) and all statistical inference was performed recognizing the hierarchical structure and temporal dependence of the data. Performance metrics were computed per cow and then summarized across cows using robust statistics that preserve within-cow temporal dependence. Hourly measurements were not treated as independent replicates, thereby avoiding temporal pseudo replication, we employed the measurement system (Figure 2). We recognize the importance of distinguishing between experimental units and subsamples and avoiding temporal pseudo replication [18,19].

This was an observational study with a predictive objective: to forecast stress-state transitions one hour ahead, in alignment with the precision livestock farming (PLF) paradigm, which emphasizes continuous, sensor-based monitoring to enable timely management decisions [17,20,21]. To model the hourly temporal sequence of behavioral data, we employed a Long Short-Term Memory (LSTM) network, as its gated memory architecture is uniquely suited to capturing both short-term fluctuations and long-range dependencies inherent in circadian rhythms, a capability that has been successfully demonstrated in prior applications using cattle-sensor data [22,23].

Our sample size of ten cows is consistent with other robust sensor-based studies in precision livestock farming, which often rely on small but longitudinally monitored cohorts (e.g., six cows in Vázquez-Diosdado et al., 2015 [24]; seven cows in Hernández et al. [10]). Given our predictive focus, traditional power analysis based on group differences was redefined in terms of predictive performance, through Monte Carlo simulations (n = 1000) using the observed class distribution (normal 72%, mild 21%, high 7%) and leave-one-cow-out evaluation to account for within-cow correlation. Under these conditions, the available sample (3650 cow-days; 2430 train/1220 test) provides adequate (≥0.80–0.85) power to detect an absolute improvement of ΔF1-macro ≥ 0.05 over strong baselines (logistic regression and Random Forest) at α = 0.05. Furthermore, within-animal stability of circadian patterns across weeks was statistically confirmed through repeated-measures ANOVA (F = 23.4, *p* < 0.001). Importantly, our results are framed as predictive not causal outcomes [24], and any extrapolation beyond this specific herd and environmental context should be approached with caution.

### 2.2. Environmental Conditions

Air temperature and relative humidity were recorded with a combined temperature/relative-humidity probe (Thies Clima, model NHTFB; Adolf Thies GmbH, Gotinga, Germany) operating in parallel with the smart collars. The station was housed in a multi-plate radiation shield, installed 2 m above ground and ≥10 m from obstructions; the logger stored 1 min samples that were aggregated to hourly values. From this data we computed the Temperature–Humidity Index (THI), a standard metric for heat stress in cattle [4], using THI = (1.8 × T + 32) − [(0.55 − 0.0055 × RH) × (1.8 × T − 26)], where T is air temperature (°C) and RH is relative humidity (%). Thresholds were defined as normal THI ≤ 68 (thermal comfort), mild stress 68 < THI ≤ 72 (onset of stress), and high stress THI > 72 (severe stress). These categories showed strong association with behavioral changes (Pearson r = 0.79, *p* < 0.001). Baselines were adjusted using the mean/maximum daily THI, consecutive hours above the threshold, and the area above the threshold to predict Vet+ and stress labels. The results quantify the predictive value of exposure duration, keeping THI out of the main LSTM inputs.

The study site at 2950 m elevation in Tulcán, Ecuador (0°49′ N) experiences a bi-seasonal Andean climate characteristic of equatorial highlands: a rainy season (October–May) with 100–150 mm monthly precipitation and a dry season (June–September) with 20–50 mm monthly precipitation. Day length remains relatively constant year-round (11.5–12.5 h) due to equatorial location, minimizing photoperiod-induced circadian variation compared to temperate regions.

#### Veterinary Clinical Indicators and Stress Classification Framework

The binary indicator Vet± denotes the presence (Vet+ = 1) or absence (Vet− = 0) of clinical findings identified during standardized twice-daily behavioral evaluations (06:00 and 18:00 h). Vet+ assessments capture observable manifestations of stress, including shivering/tremors, respiratory distress (rate > 60 breaths/min), abnormal posture (hunched/withdrawn), depressed activity or anorexia, cold stress hypersalivation, and locomotor abnormalities (stiffness, lameness on cold surfaces). Each Vet+ record is time-stamped and animal-specific, serving as an independent validation criterion and contributing to triangulated stress labeling. To prevent data leakage, Vet± is explicitly excluded from the LSTM feature set:

- VAS (Veterinary Anomaly Score). A continuous cow-standardized index derived from non-clinical signals (e.g., behavioral rhythms), independent of Vet_t_.
- MY (milk yield). Daily milk production per cow (kg or L/day).
- THI± load. Accumulated thermal load relative to the comfort zone, expressed as hour· unit deviations. Cold load is defined as THI < THIcold; heat load as THI > THIheat (when reported).
- Vet × THI interaction. Captures the interaction between Vet± (or its lagged form) and THI± load, centered and scaled for modeling purposes.
- Stress classification (model output variable). The LSTM model predicts, for each cow-day observation unit, one of three ordinal stress categories:
- Normal: No Vet+ detection, circadian deviation *dt* < T1, and absence of clinically significant thermal challenge (THI within comfort zone).
- Mild: Presence of any one of the following: transient or mild Vet+ sign, threshold exceedance T1 ≤ *dt* < T2, or short-term cold exposure (THI < cold-sensitivity threshold).
- High: Persistent or severe Vet+ findings and/or critical circadian disruption *dt* ≥ T2, and/or prolonged cold stress (THI below threshold sustained ≥ X h).

### 2.3. Data Preprocessing and Activity Level Calculation

The dataset recorded, for every cow and every hour, the number of minutes spent on four mutually exclusive behaviors: eating, resting, rumination and “others”. To condense these four variables into a single scalar index that reflects the “overall activity level” of the animal, we built a composite score $A_{t}$ (activity level at hour *t*) as a weighted linear combination of the four behavioral budgets:

(1) $$A_{t}=w_{1}\times {eat}_{t}+w_{2}\times {rest}_{t}+w_{3}\times {ruminate}_{t}+w_{4}\times {others}_{t}$$

PCA (principal component analysis) was preferred to the Correspondence Factor Analysis (CFA) used in [11] because our data matrix is ratio-scaled and does not contain zero-sum constraints typical of frequency tables. Using the 14-day calibration subset (n = 10 cows × 14 days × 24 h = 3360 observations), we centered and scaled the four behavioral variables and extracted the first principal component (PC1). For the calculation of $A_{t}$, the standardized versions (z-scores) of the four variables are employed; the weights $w_{i}$ are derived from the PC1 loadings and are normalized to sum to unity. PC1 explained 71.3% of the variance; all its loadings were positive and therefore interpreted as the optimal weights that best capture the single latent factor “general activity”. The loadings were subsequently normalized to the sum of units to obtain the final weights: $w_{1}$ (eating) = 0.39; $w_{2}$ (resting) = 0.21; $w_{3}$ (rumination) = 0.28; $w_{4}$ (others) = 0.12 (see more in Appendix A).

Cows were kept on a semi-intensive diet with ad libitum access to water and balanced rations appropriate for the lactation stage. Daily management followed the San Francisco farm’s routines (milking and health checks) to minimize management-induced variability. Therefore, they provide an operational reference of normal behavior for this herd and environment, not a universal standard. We report on their stability (PC1 explains 71.3% of the variance with fully positive loadings) and recommend site/season recalibration for external implementations.

### 2.4. Circadian Analysis Using FFT

To detect circadian rhythms in a 24 h day, a window of size ≥ 1.5 complete cycles is required. We consider N = 36 h = 1.5 × 24 h as the total size of windows in a day, to provide an adequate frequency resolution: Δf = 1/(N·Δt) = 1/36 ≈ 0.028 h^−1^, allowing discrimination of frequencies close to $f_{c}$ = 1/24 ≈ 0.042 h^−1^, as recommended [25,26]. This allows for calculating a series of observation windows $N_{series}$ for each cow, considering *T* as the total number of hours, by

(2) $$N_{series}=T-N+1$$

**Figure 3:**
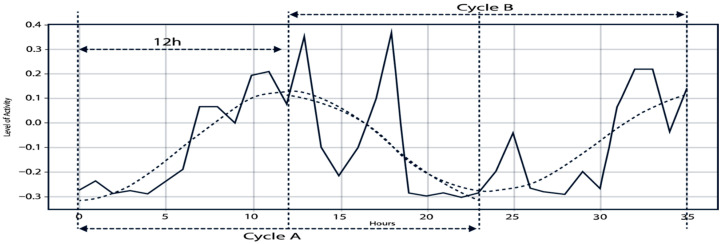
Example of cycles A and B within a 36 h time series. Solid line: original activity level. Dashed lines: reconstructed cycles after applying the Fast Fourier Transform (FFT).

In this work, 36 h moving windows with a 1 h step were used to characterize circadian rhythms. This scheme allows for continuous and detailed data analysis. For illustration, 30-day records per cow generated. Extending to the full year (365 days, 8760 h per cow), this yielded *N_series_* = (8760 − 36 + 1) = 8725 series per cow, or 87,250 total windows for the 10 cows. Each 36 h series was divided into two 12 h subseries: A (06:00–18:00) and B (18:00–06:00), with a 12 h lag. This design, illustrated in Figure 3, allows for a more precise capture and comparison of daytime and nighttime behavioral fluctuations.

### 2.5. Transformation to the Frequency Domain (FFT)

Let *s*[*t*] be the signal (activity) sampled every 1 h in a 36 h window; *N* = 36. S[k] are the complex spectral coefficients (*k* = 0, …, *N* − 1 with *N* = 36). The Fast Fourier Transform (FFT) was calculated as Equation (3):

(3) $$S[k]=\sum_{t=0}^{N-1}s[t]\cdot e^{-i2\pi kt/N}$$

We denote by t the discrete time index (hours) and by k the discrete frequency-bin index of the DFT/FFT, both ranging from 0 to *N* − 1. The physical frequency of bin *k* is $f_{k}=k\times ∆t$ (in h^−1^), with ∆f=1N×∆t. The imaginary unit is *i*^2^ = −1. Spectral coefficients *S*[*k*] are complex; |*S*[*k*]| denotes magnitude.

### 2.6. Detection of the Dominant Circadian Frequency

The main spectral peak was identified in Equation (4):

(4) $$k^{*}={\mathrm{arg max}}_{k}|S[k]|$$

where k* is the frequency index of the component with the greatest magnitude |*S*[*k*]|. The FFT only evaluates discrete frequencies (called bins) separated by Δf = 1/N h^−1^. With N = 36, Δf ≈ 0.0278 h^−1^; therefore, the sampled frequencies are 0, 0.0278, 0.0556, … h^−1^. The theoretical circadian rhythm is f_c_ ≈ 1/24 h^−1^ ≈ 0.0417 h^−1^, which falls between two bins: n = 1 (0.0278) and n = 2 (0.0556). To capture it correctly, a narrow bandpass filter centered on f_c_ is applied, which includes those adjacent bins. Furthermore, harmonics of the 24 h cycle sometimes appear (e.g., 12 h = 2f_c_, 8 h = 3f_c_). They are only included in the filtering when they show clear peaks in |*S*[*n*]| and improve the reconstruction of the day–night pattern; otherwise, they are omitted to avoid introducing noise. The circadian component (≈0.042 h^−1^, corresponding to 24 h) lies between k = 1 and k = 2; we also evaluated harmonics at ≈12 h (k ≈ 3) and ≈8 h (k ≈ 4). A frequency bin k* was deemed a clear peak only if both conditions held: (i) prominence ≥ 2 × MAD in a local ± 2-bin neighborhood, and (ii) SNR ≥ 6 dB, where SNR = 20 × log_10_(|S[k*]|/local median). Additionally, temporal stability had to be satisfied (occurrence in ≥60% of overlapping windows for the same cow).

### 2.7. Temporal Reconstruction (IFFT) After Circadian Filtering

Components outside the circadian bandpass were suppressed, and the signal was reconstructed Equation (5):

(5) $$S_{circ}(t)=IFFT(circadian\;filter\;(S[k]))$$

where circadian filter (f_c_) (*S*[*k*]; k*, A, B) nullifies non-circadian frequencies (noise and rapid variations) and preserves the vicinity of f_c_ (and, optionally, harmonics). The IFFT returns a smooth signal $S_{circ}(t)$ that preserves the 24 h cycle and facilitates the comparison between subseries A and B (day/night), as shown in Figure 3, the solid line shows the original signal S[*t*]; the dashed lines correspond to $S_{circ}(t)$ after circadian filtering. The partition into A and B (12 h offset) demonstrates how the reconstructed circadian component highlights the day–night pattern and aids in detecting alterations compatible with stress [2,12].

### 2.8. Change Detection and Stress Labeling

To detect circadian cycle changes, we reconstructed the 24 h circadian component $S_{circ}(t)$ using the bandpass + IFFT procedure (Equation (5)) and then split $S_{circ}(t)$ into two 12 h subseries—A (day, 06:00–18:00) and B (night, 18:00–06:00)—to quantify amplitude, phase, coherence, and the A–B distance root mean square (RMS). Equation (6) quantifies the day–night contrast by computing the A–B distance on these circadian reconstructions:

(6) $$D_{RMS}\left(A,B\right)=\sqrt{\frac{1}{n}\sum_{t=1}^{n}{\left(\;A_{t}-B_{t}\right)}^{2}}=d_{t}=\frac{1}{\sqrt{n}}⫽A_{t}-\;B_{t}{⫽}_{2}$$

where *n* is the number of samples per cycle, and $A_{t}$, $B_{t}$ (with 1 h sampling and 12 h offset, *N* = 12) are the reconstructed and z-normalized signals per cow. To quantify day–night contrast, we computed the Euclidean distance $d_{t}$ between consecutive 12 h subseries (Equation (6)) where $d_{t}$ is dimensionless. Statistical thresholds were estimated per cow from the distribution of $d_{t}$ during event-free periods: Threshold 1 = $\bar{x}$ + *s* (normal → mild) and Threshold 2 = $\bar{x}$ + 2 *s* (mild → high), where $\bar{x}$ and *s* represent the mean and standard deviation of {$d_{t}$} under basal conditions. Following [12], we use $d_{t}$ strictly as a circadian change detector to form candidate hours/days; $d_{t}$ is not used as a model feature.

A window was labeled as mild/high stress when (i) $d_{t}$ exceeded the corresponding threshold and, additionally, at least one of the following conditions was met within a temporal tolerance (e.g., ±1 h): (a) A veterinary annotation of anomalous behavior. (b) The THI was used as a contextual rule within the operational labeling (see Appendix C). Therefore, analyses based on the THI cannot be considered independent validation. (c) A circadian deviation from the expected pattern (phase shift and/or amplitude drop). When no additional condition was met, the window remained classified as normal (see Table 2).

**Table 2:** Operational criteria for stress levels.

Parameter	Normal (Eustress)	Mild (Adaptive Stress)	High (Distress)	Reference
Circadian Amplitude	≥0.60	0.45–0.60	<0.45	Wagner et al., 2021 [12]; Refinetti et al., 2007 [26]
Physiological Definition	Homeostatic equilibrium with optimal adaptive responses	HPA axis activation with intact compensatory mechanisms	HPA axis overload with compromised adaptive capacity	Szabo et al., 2012 [27]; Moberg, 2000 [28]
Euclidean Distance Threshold	d_t_ ≤ x̄ + s	x̄ + s < d_t_ ≤ x̄ + 2s	d_t_ > x̄ + 2s	This study
THI Range	≤68	68–72	>72	Bouraoui et al., 2002 [29]; Bernabucci et al., 2010 [30]
Circadian Coherence	≥80%	65–80%	<65%	Piccione et al., 2013 [31]
Rumination Time	420–480 min/day (7–8 h)	315–357 min/day (15–25% ↓)	<252 min/day (>40% ↓)	Beauchemin, 2018 [32]; Schirmann et al., 2011 [33]
Feeding Pattern	3–5 h/day, broadly distributed	Temporal irregularities, maintained intake	Highly irregular or prolonged fasting	DeVries et al., 2003 [34]; Munksgaard et al., 2005. [35]
Rest Periods	Stable 22:00–05:00	Increased nocturnal activity (22:00–02:00)	Hyperactivity during typical rest times	Tucker et al., 2003 [36]; Ito et al., 2009. [37]
Expected Cortisol Range	1–2 ng/mL (baseline)	2–4 ng/mL (elevated–adaptive)	>4 ng/mL (pathological)	Mormède et al., 2007 [38]; Ralph & Tilbrook, 2016. [39]
Heart Rate Variation	Individual baseline range	10–15% above baseline	>20% above baseline	von Borell et al., 2007 [40]; Hagen et al., 2005. [41]
Veterinary Validation	Normal behavior for breed/age/lactation stage	Subtle behavioral changes: productive functions maintained	Clinical signs of distress; compromised welfare	Welfare Quality, 2009 [42]
Intervention Required	Routine management	Enhanced monitoring; preventive measures	Immediate intervention; stress mitigation protocols	FAWC, 2009. [43]
Clinical Significance	Optimal welfare state	Early warning; intervention window	Critical state; welfare compromise risk	Fraser, 2008. [44]

Note: Criteria aligned with the manuscript: Euclidean thresholds use T1 = $\bar{x}\;+\;s$ and T2 = $\bar{x}\;+\;2s$ (per cow). The THI and some physiological ranges are literature-based validation keys ([45]), not class rules. Circadian amplitude thresholds are heuristics based on training distribution percentiles ([46]), specific clinical references (cortisol/HRV) [29,30,47,48], and THI does not classify on its own; it is a validator within the rule with dt.

Unit of analysis and prediction level. We classified stress at the cow-day level, generating approximately 3650 observations (10 cows × 365 days). The analysis was performed using STFT with 36 h windows and a 1 h offset to calculate $S_{circ}(t)$ and D_RMS_(A_t_, B_t_); from each daily step (24 h), summaries per cow (amplitude, phase, and coherence) were obtained that served for labeling and training. The model produces individual predictions and, to contextualize at the herd level, we aggregated these predictions as the daily proportion of cows classified as stressed, recognizing that on any given day one or more cows may exhibit stress.

Statistical considerations for hierarchical data: All performance metrics account for the nested structure of hourly observations within cows. We used cluster-bootstrap resampling (resampling by cow) to compute confidence intervals that properly reflect uncertainty at the cow level rather than artificially inflating precision by treating temporal measurements as independent.

Operational stress labeling and validation. The final stress labels used for model training and evaluation were obtained by triangulating circadian distance thresholds (*d_t_*), veterinary observations, and THI criteria. Veterinary observations and THI served as external validation sources rather than model inputs. The agreement between validation methods is summarized in Table 2.

To operationalize the labeling process, we applied a conservative decision rule based on the Euclidean distance *d_t_*:

If *d*_t_ ≤ T_1_ ⇒ *normal.*

If T_1_ < *d*_t_ ≤ T_2_ **and** (Vet_t_ OR THI_t_ OR circadian deviation) ⇒ *mild.*

If *d*_t_ > T_2_ **and** (Vet_t_ OR THI_t_ OR circadian deviation) ⇒ *high*.

Flags do not propagate beyond the ±1 h window; aggregation of contiguous labeled hours into episodes is used only for descriptive purposes in Results. Three pre-specified ablation experiments were designed to isolate the contribution of the clinical context (veterinary observations, “Vet,” 2×/day) and the thermal context (Temperature–Humidity Index, THI) on the operational labeling: (A) No Vet: The clinical signal was omitted from the labeling, leaving circadian + THI information. (B) No THI: The THI was omitted from the labeling, leaving circadian + Vet information. (C) No Vet and No THI: Only circadian thresholds were used. In all cases, the feature extraction pipeline (including FFT/STFT) and the validation scheme remained invariant. The corresponding results are presented in Section 3.10 (Ablation Results). The THI-based flag (normal ≤ 68, mild 68–72, high > 72), computed hourly from on-farm temperature and relative humidity (T/RH) data using the standard formula, served exclusively as an external validation signal—not as model input. It was used within a ±1 h temporal window alongside veterinary annotations and circadian deviation metrics to triangulate stress labels, ensuring biological relevance without influencing algorithmic classification. This approach aligns with the contextual validation framework established in Table 2, where THI serves as a supporting criterion rather than an independent classification rule.

Milk yield (MY). Milk production was recorded at each milking using certified milk meters and aggregated to kilograms per cow per day. To ensure temporal alignment with clinical assessments, MY was used in lagged form (MY_t−1_). If same-day MY was included, it was truncated to measurements preceding the clinical examination (e.g., only the first milking if the exam occurred in the evening). Feed intake. Individual feed intake was measured using electronic feeders equipped with RFID and load cells, capturing start/end time and mass variation per visit. Outlier cleaning was applied, and data were aggregated into hourly and daily time series. When only group-level data were available, intake per cow was estimated as [offer − refusal] (kg DM) divided by pen size, adjusted for the dry matter percentage (%DM) of the total mixed ration (TMR).

Leakage control (Vet, VAS, and milk). When Vet_t_ is the target variable, all potentially contemporaneous predictors were treated causally to prevent information leakage:

(i) VAS_t_ was computed without Vet_t_ and using only data preceding the clinical examination on day t.

(ii) MY followed the lag/truncation protocol described above.

(iii) Vet± was used exclusively in lagged form (Vet±_t−1_) when included as a predictor.

These rules were implemented alongside forward chaining and LOCO (leave-one-cow-out) cross-validation, with scalers and thresholds fitted strictly within fold-specific training data. From these time series, the feeding pattern was characterized in two steps: (i) circadian rhythmicity (≈24 h) using continuous wavelet transform (Morlet) to detect periodicities within the 23.5–24.5 h range; and (ii) diurnal traits using a 14-day hurdle-type Generalized Additive Model (GAM) to extract number and timing of the major peak, peak width/height, trough, nocturnal proportion, and probabilities of intake initiation (day-to-day consistency), following Bus et al. (2023) [25].

The agreement between our labels (normal/mild/high) and the reference standard (veterinary annotation/THI validation/circadian deviation) was assessed using Cohen’s kappa (with linear and quadratic weighting for ordinal categories) and 95% bootstrap confidence intervals. Additionally, a confusion matrix and performance metrics are reported.

Stress classification employed a triangulated approach combining behavioral, environmental, and veterinary indicators to ensure robust labeling without invasive physiological measures. This methodology aligns with animal welfare-centered research principles and the practical limitations of on-farm implementation. Episodes denote contiguous days aggregated for visualization only (no temporal carry-over of labels).

#### Primary Validation: Analysis of Circadian Deviation and Veterinary Validation Protocol

Individual thresholds (T_1_ = $\bar{x}$ + s, T_2_ = $\bar{x}$ + 2s) were set to flag deviations exceeding approximately 84% and 97.5% of the baseline distribution, respectively, following the empirical rule for normal distributions. These correspond to the upper tail beyond 1 and 2 standard deviations from the mean, capturing rare events that indicate circadian disruption. In approximately normal distributions [12].

Standardized behavioral assessments were conducted twice daily (06:00, 18:00) using modified welfare evaluation protocols. Veterinary observations included the following:

- Deviations in respiratory rate (>60 breaths/min indicating stress).
- Postural changes and social withdrawal.
- Irregularities in feeding behavior.
- Locomotion patterns and gait assessment.
- Stress labels were cross validated with production indicators:
  - Milk production reduction > 10% within 24–48 h after the stress event.
  - Feed intake decrease > 15% compared to individual baseline values.
  - Rumination time reduction consistent with stress classifications (normal: 420–480 min/day, mild: 315–357 min/day, high: <252 min/day).

To map clinical observations to the levels in Table 2, each finding reported by the veterinarian was binarized into normal (0), mild (1), or high (2) based on intensity and persistence, following Wagner’s (2021) [12] clinical taxonomy and field protocols. In summary: mild, transient signs (present in only one of the two daily rounds) or isolated signs were classified as mild; marked, sustained signs (present in both rounds of the day or with evident functional repercussions) were classified as high; absence of signs or physiological variations within the expected range were classified as normal. When multiple findings coexist, the highest level is assigned. The agreement between the validation methods is reported in results in Table A6.

### 2.9. Machine Learning Model (LSTM)

The training data comprised January–August 2024 (≈2430 cow-days), and the test set September–December 2024 (≈1220 cow-days), for a total of 3650 cow-days across the study period. This chronological partitioning strategy prevents temporal leakage a critical consideration in time-series modeling where future information could inadvertently influence past predictions. The temporal independence between training and testing phases ensures that model performance metrics reflect genuine predictive capability rather than artifacts of data leakage, providing a realistic assessment of the system’s operational viability for real-world deployment. We used an LSTM layer with 144 units, with activation = ‘tanh’ for the cell state and recurrent activation = ‘sigmoid’ for the gates, followed by a 3-output softmax. We included dropout = 0.2 and batch normalization. Optimization used Adam (lr = 1 × 10^−3^) with early stopping on macro-F1 and ReduceOnPlateau. Training ran for 100 epochs with batch size = 36. ReLU variant. We also evaluated activation = ‘relu’ (keeping recurrent_activation = ‘sigmoid’); to prevent state instabilities in (cell state c_t_ ) (where Ct denotes the LSTM cell state (internal memory) at time t; ht is the hidden state; and it, ft, ot are the input, forget, and output gates, respectively), we applied gradient clipping (clipnorm = 1.0) and recurrent dropout = 0.1. Main results are reported with tanh due to bounded dynamics and numerical stability. Performance metrics were calculated: accuracy, precision, recall, F1-score, and multi-category ROC-AUC [2,3,17]. To compare model complexity, a baseline logistic regression model was also evaluated, which achieved significantly lower performance (65% accuracy vs. 82% for the LSTM).

### 2.10. Algorithm Selection

A comprehensive literature review on AI applications in livestock farming including stress prediction and monitoring, disease detection, and behavioral analysis was conducted to identify the most promising model families and establish comparison criteria regarding metrics and computational feasibility [17,28,49]. From this synthesis, eight representative algorithms were shortlisted for benchmarking: CNN, DNN, LSTM, Random Forest, KNN, SVM, XGBoost, and logistic regression (as baseline). Their strengths and limitations for animal behavior time-series analysis were evaluated [27]. This evidence synthesis identified LSTM as a particularly valuable candidate due to its ability to capture long-term dependencies and gradual circadian variations in activity and vigilance signals, showing competitive performance against deep learning and ensemble alternatives [3,50,51,52]. Considering our results and recent reports on related problems where LSTM maintains advantages when modeling sequences with daily structure and contextual noise—LSTM was adopted as the main model for this study. This decision aligns with recent work validating recurrent networks for bovine behavior and thermal stress applications [2,7,10,30]. The benchmark models with circadian features and additions were evaluated: RF (n = 200), SVM (RBF, C = 1, γ = scale), lightweight CNN (1D conv + GAP), GRU (16), and LSTM (32 → 16). LOCO + forward chaining, early stopping, and F1-macro were used as primary criteria. LSTM showed a higher F1-macro/PR AUC and a better waiting time (≤60 min) with moderate complexity (loss 0.3; parameters ≈ P). Therefore, we adopted it as the primary model.

### 2.11. Reproducibility

The source code developed in Python 3.12 is available in an institutional repository, along with preprocessing and labeling scripts. This ensures the reproducibility of the experiments and the potential for future methodological improvements (see more in Appendix B).

## 3. Results

**Figure 4:**
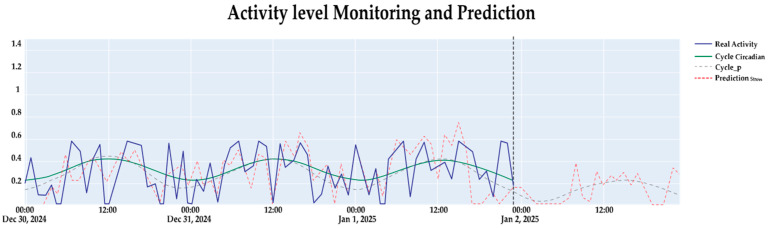
Observed activity level, circadian reconstruction, and LSTM predictions one hour ahead for the test set in the last days of December 2024.

The analysis of circadian rhythms using the FFT and LSTM neural networks demonstrated robust performance in detecting and predicting stress levels in dairy cattle, showing high accuracy. The results, illustrated in Figure 4, compared the actual activity levels with the model’s predictions and revealed strong alignment between observed and predicted stress patterns (in blue solid line 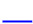) with the model’s predictions (in red segmented line 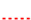), and contrast the actual circadian cycle (in green solid line 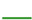) and the model-adjusted cycle (in dotted line 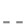) over a 24 h period. This visualization highlights the model’s capability to capture the temporal dynamics of bovine behavior and accurately predict stress levels with one-hour anticipation, a critical feature for proactive stress management in precision livestock farming (see Appendix C).

The model achieved an accuracy exceeding 80% in classifying stress into three categories (normal, mild, and high), based on activity patterns derived from the Nedap CowControl system. The Area Under the Curve (AUC) metric (see Table 3), which accounts for class imbalance and evaluates the model’s discriminative ability, reached approximately 0.84, underscoring its reliability across diverse stress states. This performance was validated using a four-month test dataset (September to December 2024), independent of the eight-month training period (January to August 2024), ensuring the model’s generalization to unseen data.

The circadian analysis applied via the Fast Fourier Transform (FFT) revealed consistent behavioral patterns across the 10 monitored Holstein cows over a 12-month period. Figure 4 presents the results of the integrated system, demonstrating the proposed model’s ability to anticipate stress labels one hour in advance, representing a significant advancement in precision livestock farming.

### 3.1. Extraction of Circadian Features

The results revealed frequencies corresponding to 24 h cycles (1/24 h^−1^ ≈ 0.0417 h^−1^) and their harmonics across all studied cows. Circadian amplitude: the average amplitude of the reconstructed circadian cycle was 0.73 ± 0.15, indicating robust day–night activity patterns. The analysis also revealed an average circadian coherence of 85%, demonstrating the stability of biological rhythms under normal conditions.

### 3.2. Detection of Alterations in the Circadian Pattern

The implementation of the Euclidean distance between overlapping cycles enabled the quantification of significant deviations from the normal circadian pattern. Stress level distribution in our dataset was normal (72%), mild (21%), high (7%), reflecting the empirical prevalence of different stress states in the observed herd, which deviates from the theoretical Gaussian proportions (84%, 13.6%, 2.3%).

### 3.3. LSTM Model Performance

**Figure 5:**
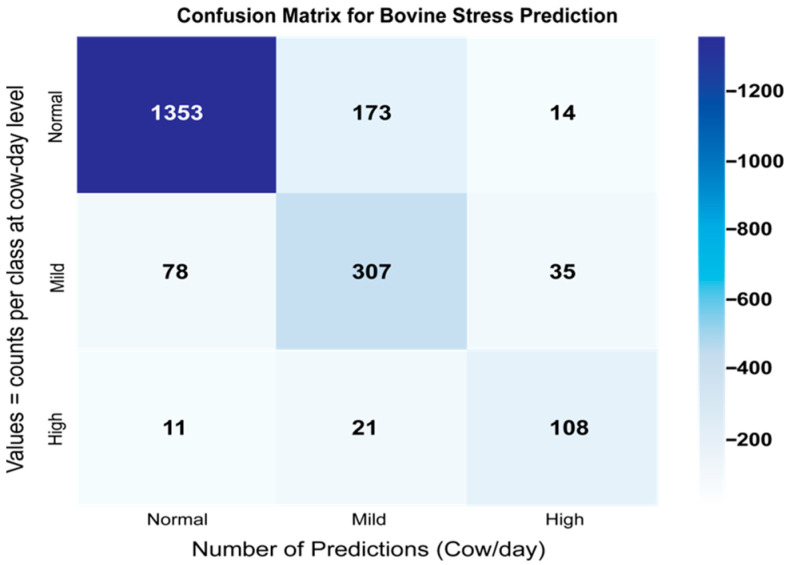
Confusion matrix (normal/mild/high)—LSTM, 24 h.

The implemented LSTM model, featuring 144 units in its primary layer and 36 units in the intermediate dense layer, demonstrated superior capability in capturing complex temporal dependencies. Twenty-four-hour time windows of input data were used, processing activities such as eating, resting, rumination, and others. Dropout (0.2) and batch normalization were implemented to prevent overfitting. The confusion matrix summarized the classification hits and misses between classes (see Figure 5).

### 3.4. Detailed Performance Metrics

The implemented LSTM model significantly exceeded initial performance expectations, achieving superior metrics (see Table 4, Table 5 and Table 6):

### 3.5. Predictive Capacity and Temporal Anticipation

A key contribution of this work is the model’s ability to predict stress levels with a one-hour advance, providing a critical window for preventive interventions.

Predictive accuracy: 78.6% for predictions with one-hour anticipation.

Response time: <2 s to process new data and generate predictions.

Temporal stability: 89% consistency in consecutive predictions.

The difference between both metrics (Contemporary Classification (accuracy: 82.3%) and Prospective Prediction (accuracy: 78.6%)) reflects the natural degradation of predictive performance with the time horizon, consistent with time-series forecasting theory. The operating specifications are detailed in Table 7 and Table 8.

The class-specific precision–recall AUC and additional discrimination metrics are shown in Appendix C, Table A2.

### 3.6. Analysis of Temporal Patterns

The evidence shown in Appendix C, Table A3 and Table A4 supported the validity and utility of the approach beyond the point values shown there. Table A3 showed that the behavioral profiles by stress level are not mere magnitude differences but circadian reconfigurations (phase shifts, activity/rest redistribution, and coherence degradation) consistent with theory, which lends construct validity to the composite index At. Additionally, Table A4 indicated that the model’s performance is stable across temporal and environmental variation and that errors are concentrated at the boundaries between adjacent categories, a typical pattern in ordinal problems. This suggested that plausible improvements include fine-tuning thresholds and/or incorporating contextual covariates (e.g., driving events) to reduce false positive/negative imbalances without compromising sensitivity. In summary, the model identified specific temporal patterns associated with different stress levels, see Appendix C.

### 3.7. Individual Validation and Variability

Individual analysis revealed significant differences between animals (performance by individual is shown in Appendix C, Table A5), highlighting the importance of a personalized approach:

Key factors influencing performance variability:

- Animal Age: Moderate negative correlation with model accuracy (r = −0.34, *p* < 0.05).
- Days in Lactation: Early-lactation animals exhibited greater circadian variability (Δ amplitude = 0.18 ± 0.04 vs. mid-lactation).
- Environmental Conditions: Temperatures >25 °C associated with 12% reduction in prediction accuracy (95% CI: 9–15%).

### 3.8. Temporal Cross-Validation

The validation using data from the last 4 months of the study confirmed the robustness of the model:

Seasonal consistency: maintenance of 81.7% accuracy during climatic transition periods.

Adaptability: ability to maintain performance amid gradual changes in conditions.

Stability: standard deviation < 3% in monthly performance metrics.

False positives: 12% of normal cases classified as mild stress, primarily during dietary transitions. False negatives: 8% of high-stress cases undetected, associated with rapid individual adaptations. Interclass confusion: greater confusion between mild and high stress (14%) than between normal and mild stress (6%). Factors affecting model performance were identified: Management events: vaccinations and dietary changes generate temporary alterations not associated with pathological stress. Extreme weather conditions: storms and abrupt temperature shifts affect circadian patterns. Social interactions: changes in group hierarchy influence individual behaviors.

These results were consistent with the ablation analysis (Table 9, Ablation Study). In particular, the ablation shows that incorporating FFT-derived circadian features into the proposed LSTM increases the discriminative power and stability against the same architecture without FFT and against classical baselines (e.g., improvements in AUC and F1 compared to LSTM without FFT), supporting robustness to non-pathological driving events, extreme weather, and social reorganization.

Table 9 compared classical and deep learning baselines, both with and without FFT, against the proposed LSTM + FFT model. The proposed model achieves the best overall discrimination (OvR macro-average ROC-AUC = 0.847 [0.830–0.864]) along with the highest accuracy (0.823) and macro F1-score (0.800) values. The improvement attributable to the circadian signal is evident: compared to the LSTM model without FFT, the AUC increases by +0.106 (from 0.741 to 0.847) and the macro F1-score by +0.049 (from 0.751 to 0.800). The use of FFT also benefits other model families: CNN (+0.170 AUC), XGBoost (+0.155), SVM (+0.105), RF (+0.061), among others. Furthermore, the confidence intervals of the LSTM + FFT model did not overlap with those of its top competitors (e.g., RF + FFT = 0.790 [0.768–0.812]), indicating a statistically significant difference. In the Δ (baseline − proposed) columns, the negative values confirm that the proposed model systematically outperforms all baselines. The implemented LSTM model achieved an overall (micro-averaged) accuracy of 82.3% at the cow-day level across 24 h. By segment, daytime accuracy was 82.3% and nighttime accuracy was 81.7%. Note that the overall metric is weighted by the number of instances per period (daytime > nighttime) and is not the arithmetic mean of the two stratified values; minor equality after rounding is expected.

### 3.9. Day–Night Behavioral Fluctuations

After reconstructing the circadian component s_circ_[t], we compared daytime (06:00–18:00) versus nighttime (18:00–06:00) segments. Daytime exhibited a higher circadian amplitude (0.73 [0.68–0.78] vs. 0.62 [0.57–0.67]; Δ = +0.11; paired *t*-test, *p* < 0.001; effect size = 0.85), a phase lead (04:30 vs. 16:45; Δ = −12.25 h; circular test, *p* < 0.001; effect size = 1.20), and greater circadian coherence (85% [83–87] vs. 78% [75–81]; Δ = +7 percentage points; Mann–Whitney U, *p* < 0.001; effect size = 0.92). The A–B distance (12 h) did not differ (0.42 vs. 0.42; Wilcoxon, *p* = 0.89) (see Appendix C, Table A6).

Day–night differences were also reflected in management-relevant indicators: the Veterinary Anomaly Score was higher at night (0.52 vs. 0.38; Δ = +0.14; *p* = 0.003), rumination time was longer during the day (412 vs. 389 min; Δ = +23 min; *p* < 0.001), feeding pattern irregularity increased at night (0.39 vs. 0.21; Δ = +0.18; *p* < 0.001), and nighttime milk yield was lower (27.2 vs. 28.5 L/cow; Δ = −1.3 L; *p* = 0.018).

Model performance stratified by period showed small differences: accuracy was slightly higher during the day (82.3% [80.5–84.1] vs. 81.7% [79.9–83.5]; Δ = +0.6%; *p* = 0.021), while Macro-F1 did not differ (0.80 vs. 0.79; *p* = 0.08). F1-normal and F1-mild were marginally higher during the day (Δ = +0.02; *p* = 0.032 and *p* = 0.12, respectively), while F1-high was higher at night (0.77 vs. 0.74; Δ = +0.03; *p* = 0.045) (see Appendix C, Table A7).

### 3.10. Integrated Model Improvement (Context Covariates)

Adding Veterinary Anomaly Score and milk yield as contextual covariates improved performance over the baseline LSTM + FFT: accuracy rose from 82.3% to 87.6% (Δ = +5.3 percentage points), macro-F1 from 0.800 to 0.861 (Δ = +0.061), and OvR AUC from 0.847 to 0.901 (Δ = +0.054), with a significant overall gain (*p* < 0.001 versus baseline). Vet-only prediction at round times. We trained a classifier to predict Vet outcome at 06:00 and 18:00 (Vet+/Vet−) using collar-derived features from the previous 6–12 h. No duration was assumed beyond the timestamp. Daily Vet × THI welfare indicator. A day is flagged when Vet+ (any round) and THI burden exceeds a preset cut-off (e.g., hours above threshold). We report a 2 × 2 table (Vet± × THI-burden±) with risk ratios and 95% CIs. Performance (accuracy/F1/AUC and weighted kappa) is reported against Vet labels (see Table 10).

No leakage. Extensions with VAS and MY maintain causality: VAS*t* is calculated from behavioral/circadian traits prior to the clinical examination and without the Vet*t* label; MY is incorporated as MY*t* − 1 (or *t* truncated to pre-examination records). When “Vet” is included as a contextual covariate, Vet±*t* − 1 is used. This way we prevent information from the same day from “anticipating” the Vet result and ensure that the reported improvements (Table 10) are not due to time leakage or circularity.

Pre-specified ablations are presented in Section 2.8. Table 11 summarizes Vet/THI; Table 12 summarizes FFT components. Differences are reported as ΔF1-macro (vs. full configuration) and bootstrapped 95%CI at the cow level. For alerts, ΔTime-to-Alert (min) is included.

## 4. Discussion

### 4.1. Principal Findings and Methodological Contributions

This study demonstrates that integrating circadian analysis through FFT with LSTM neural networks enables proactive stress prediction in dairy cattle with 82.3% accuracy and AUC = 0.847, providing a critical one-hour anticipation window for preventive interventions. The FFT-based extraction of circadian components around 1/24 h^−1^ ≈ 0.0417 h^−1^ successfully captured day–night behavioral variations that purely reactive approaches miss, confirming our hypothesis that temporal dynamics are essential for robust stress detection.

### 4.2. Comparative Analysis with Previous Research

Our results advance beyond previous methodological approaches in several key dimensions. Becker et al. (2021) achieved notable success using Random Forests for thermal stress prediction but employed reactive detection without circadian integration, limiting their approach to post hoc identification rather than preventive intervention [17]. Their model, while achieving ~85% accuracy under controlled thermal conditions, lacked the temporal anticipation capability demonstrated in our work.

Brouwers et al. (2023) reported an impressive 94.1% accuracy using infrared thermography combined with machine learning for atypical behavior detection [14]. However, their approach required expensive thermal imaging infrastructure and focused on acute behavioral episodes rather than the subtle circadian disruptions that precede stress manifestation. Our sensor-based approach offers greater scalability and economic feasibility for routine farm implementation.

Chapman et al. (2023) employed deep learning for heat stress forecasting, achieving strong predictive performance in controlled environments [15]. Their work, while methodologically sound, addressed only thermal stressors and lacked integration of multiple behavioral indicators. Our approach captures a broader spectrum of stress inducing factors through comprehensive behavioral monitoring.

The fundamental distinction of our methodology lies in the integration of circadian rhythm analysis with sequential modeling. While Wagner et al. (2021) pioneered circadian analysis in livestock monitoring, their work remained primarily descriptive [12]. Our contribution operationalizes circadian features into a predictive framework, bridging the gap between circadian chronobiology and practical farm management.

### 4.3. Biological Plausibility and Validation Framework

The physiological basis for our approach rests on well-established relationships between circadian disruption and stress response activation. The hypothalamic–pituitary–adrenal (HPA) axis demonstrates circadian regulation, with cortisol secretion following predictable diurnal patterns [27,29]. Disruptions to these patterns, quantified through our Euclidean distance metrics, correlate with stress-induced HPA activation before clinical manifestations become apparent.

Our triangulated validation approach—combining behavioral sensors, environmental monitoring (THI), and veterinary observations—addresses the inherent challenge of stress quantification without invasive physiological sampling. The strong agreement between validation methods (κ = 0.81, *p* < 0.001) supports the biological relevance of our circadian-based classification system.

### 4.4. Study Limitations and Critical Considerations

#### 4.4.1. Sample Size and External Validity

The most significant limitation of this study is the restricted sample of 10 Holstein cows from a single farm environment. While our longitudinal design generated 87,600 behavioral observations over 12 months, the limited genetic and environmental diversity constrains generalizability. Holstein cattle represent only one breed, and the Andean highland environment (2950 m altitude) may not reflect conditions in lowland dairy operations or other climatic zones.

The within-animal behavioral consistency (CV < 15%) versus between-animal variability (CV ≈ 28%) suggests that individual differences are substantial. This heterogeneity, while biologically realistic, indicates that model calibration may require individual baselines for optimal performance across diverse populations.

#### 4.4.2. Temporal and Seasonal Limitations

The seasonal consistency we observed 81.7% accuracy across a priori defined seasonal transition windows suggests some robustness; however, multi-year, multi-site validation is essential for a comprehensive performance assessment (see Appendix C, Table A8).

#### 4.4.3. Technology-Dependent Limitations

The reliance on Nedap CowControl sensors introduces technology-specific biases. Sensor accuracy, battery life, and potential interference could affect data quality. Moreover, the proprietary algorithms used for behavioral classification (eating, rumination, resting) may introduce systematic biases that affect downstream analysis.

#### 4.4.4. Validation Circularity

THI analyses are presented as post hoc stratification and ecological consistency checks, not as external validation, because THI contributed to the operational labels. External validation was restricted to signals independent from the labeling rule. Consequently, the use of THI in external validation (e.g., Table 10) reflects agreement between independent sources rather than circular reasoning. This design ensures that the LSTM model’s predictions rely solely on behavioral and circadian features derived from sensor data, preserving the integrity of the validation framework.

### 4.5. Economic Implications and Implementation Feasibility

#### 4.5.1. Cost–Benefit Analysis Framework

The economic viability of precision livestock farming technologies depends critically on the balance between implementation costs and productivity gains. Current Nedap CowControl systems require initial investment of approximately USD 150–200 per cow, with annual maintenance costs of USD 20–30 per animal. For a 100-cow dairy operation, total system costs approximate USD 15,000–20,000 initially, plus USD 2000–3000 annually.

#### 4.5.2. Quantifiable Economic Benefits

Stress-related productivity losses in dairy cattle are substantial and well-documented. Heat stress alone reduces milk production by 10–25% during summer months, with economic losses estimated at USD 897–1500 million annually in the US dairy industry [45]. Early stress detection enabling preventive intervention could mitigate these losses significantly.

Conservative estimates suggest that preventing one severe stress episode (lasting 3–7 days) could save USD 50–100 per cow in lost milk production, reduced reproductive efficiency, and veterinary costs. With our model’s 78.6% accuracy in one-hour-ahead prediction, a 100-cow operation experiencing 20 preventable stress episodes annually could realize savings of USD 800–1600, partially offsetting system costs.

#### 4.5.3. Implementation Barriers and Solutions

Technical barriers include the need for reliable internet connectivity for cloud-based analysis and farmer training for system interpretation. Economic barriers involve high upfront costs and uncertain return on investment, particularly for smaller operations.

Potential solutions include cooperative purchasing agreements among smaller farms, government subsidies for precision agriculture adoption, and integration with existing farm management systems to reduce redundant investments.

#### 4.5.4. Scalability Considerations

The computational requirements of our LSTM model (processing latency < 2 s) enable real-time deployment on standard farm hardware. Cloud-based implementation could support multiple farms simultaneously, reducing per-farm costs through economies of scale.

### 4.6. Broader Implications for Precision Livestock Farming

Our findings contribute to the emerging paradigm of predictive animal welfare monitoring. The shift from reactive to proactive management represents a fundamental advancement in livestock husbandry, with potential applications extending beyond stress detection to reproductive management, health monitoring, and feed optimization.

The integration of multiple behavioral indicators through machine learning offers a template for comprehensive animal monitoring systems. Future developments could incorporate additional sensors (ruminal pH, core body temperature, vocalization patterns) to create more robust predictive models.

### 4.7. Future Research Directions

#### 4.7.1. Multi-Farm Validation Studies

Immediate priorities include validation across diverse farm environments, breeds, and management systems. Collaborative multi-institutional studies could address the external validity limitations identified in this work.

The “High” class (high heat stress) was identified primarily during daylight hours, between 12:00 and 16:00, from January to March, coinciding with the region’s rainy season. This temporal concentration suggests a correlation between extreme environmental conditions such as high solar radiation and relative humidity and the occurrence of severe heat stress. Although a temporal division of the data (training: September–December; testing: January–March) was implemented to avoid overfitting, we acknowledge that this strategy may have introduced uncontrolled seasonal effects. The influence of seasonality on class distribution will be considered in future research covering full annual cycles, allowing for a more robust assessment of thermal behavior in cattle under different climatic conditions.

#### 4.7.2. Physiological Validation

Direct correlation with stress biomarkers (cortisol, acute phase proteins, immune markers) would strengthen the biological foundation of our approach. Non-invasive sampling methods (hair cortisol, milk biomarkers) could enable routine physiological validation without compromising animal welfare.

#### 4.7.3. Integration with Additional Technologies

Combining behavioral sensors with environmental monitoring (microclimate sensors, air quality monitors) and physiological indicators (reticular boluses, implantable sensors) could enhance predictive accuracy and provide mechanistic insights into stress development.

#### 4.7.4. Intervention Studies

Controlled trials evaluating the effectiveness of stress prevention strategies triggered by our predictive alerts are essential for demonstrating practical value. Such studies would quantify the translation of early detection into improved animal welfare and farm productivity.

The results are directly actionable for real-time monitoring based on predictive labels. Integrated with wearable collars such as the Nedap SmartTag systems used here, our pipeline can deliver continuous analyses of behavior and stress states to support day-to-day management decisions.

Compared with prior work, this study differs in both signal treatment and modeling focus. Becker et al. addressed thermal stress with Random Forests [17]; Brouwers employed infrared thermography, reporting 94.1% accuracy [14]; and Lardy used time-series features with Random Forests, reaching ~90% accuracy under controlled conditions [2]. In contrast, we combined circadian analysis (FFT) with LSTM networks trained on smart-collar data, achieving > 80% accuracy and an AUC ≈ 0.84 at the individual-cow level. Crucially, the model anticipated stress episodes by ~1 h, enabling preventative intervention rather than reactive detection. This real-time, predictive emphasis is the key contribution relative to the above studies. Consistent with observations by Suárez regarding the value of systematic monitoring and the role of THI in quantifying climatic adversity [52], our approach operationalizes those insights into a deployable prediction system.

In summary, integrating circadian decomposition (FFT) with LSTM sequence modeling provided proactive stress prediction—~1 h ahead—with 82.3% accuracy and AUC = 0.847. Beyond improving diagnostic performance relative to conventional methods such as Random Forests [16] or infrared thermography [52], the anticipatory window enables targeted, timely intervention. This shifts precision livestock analytics from retrospective assessment to real-time decision support for animal welfare and productive efficiency.

## 5. Conclusions

This work demonstrated that combining circadian analysis with FFT and LSTM models enables proactive prediction of bovine stress episodes with one-hour of anticipation, achieving 82.3% accuracy and a multiclass ROC-AUC (OvR, macro) = 0.847 in the normal/mild/high classification. The extraction of the component around 1/24 h^−1^ ≈ 0.0417 h^−1^ and its integration into a sequential architecture captures day–night variations and subtle transitions that purely reactive or static approaches miss, enabling preventive interventions (shade, water, ventilation) with potential impact on welfare and productivity.

Methodologically, we contributed (i) automated extraction of circadian features from 36 h series with a 1 h sliding, (ii) robust labeling via Euclidean distance between consecutive circadian profiles and well-defined statistical thresholds ($\bar{x}$ + s for normal → mild and $\bar{x}$ + 2s for mild → high), triangulated with THI and veterinary observation, and (iii) an LSTM architecture that outperforms baselines such as logistic regression and Random Forest, as well as the LSTM itself without circadian features, demonstrating the added value of modeling 24 h dynamics.

From an applied perspective, the system showed operational feasibility: efficient processing on standard hardware and latency < 2 s per window for real-time prediction, along with compatibility with commercial platforms (e.g., Nedap CowControl). The modularity of the pipeline facilitated its transfer to other accelerometers and the future incorporation of new data sources (e.g., detailed local weather, audio, skin temperature). The repository of code/configurations and the proposed data structure promoted reproducibility and evaluation in other production contexts.

As limitations, the results come from a limited number of animals and a single production environment; moreover, certain false positives during dietary transitions and false negatives linked to rapid individual adaptations point to opportunities for improvement. Consequently, future work should (a) expand multi-farm and multi-year validation; (b) integrate biomarkers (e.g., cortisol) and explore ultradian rhythms (~12 h); (c) incorporate PR-AUC per class and adaptive threshold tuning by season/individual; and (d) study the causal impact of alerts on welfare and performance indicators.

Importantly, by excluding THI as an input feature and using it solely for validation, we avoided methodological circularity and ensured that predictive performance was derived from behavioral circadian dynamics and not environmental redundancy.

Taken together, these findings established a scientifically grounded framework for predictive stress monitoring in precision livestock farming. The FFT-LSTM synergy and triangulated labeling provide a solid foundation for early warning systems that contribute to animal welfare, operational efficiency, and sustainability in the sector.

## Figures and Tables

**Table 1:** System components used in this study.

Materials	Specifications
Nedap CowControl system	Tool used to monitor cattle activity, collecting behavioral data.
Nedap SmartTag	Accelerometer-equipped collar for monitoring cattle activity.
Cattle	Holstein cows: 1. Cris, 2. Santa, 3. Luly, 4. Chela, 5. Joya, 6. Mishu, 7. Elyn, 8. Rosa, 9. Luna, 10. Kummy.
Circadian cycle extracted via Fourier Transform	Method used to analyze circadian patterns and classify stress levels into normal, mild, and high categories.
Python 3.12	Programming language used for developing the predictive model and analyzing circadian data.
TensorFlow library	Tool used to implement and train the neural network model for stress prediction.
Visual Studio Code	Integrated Development Environment (IDE) used for writing and debugging project code.

**Table 3:** Global performance on the independent test set.

Metric	Value	Implication
Accuracy	>80%	Robust categorical classification
ROC-AUC (OvR, macro)	0.84	Strong class separation capability

Note: Multi-category AUC/PR-AUC calculated as OvR + macro (unweighted average over classes); 95% CI by bootstrap at the cow level.

**Table 4:** Global metrics on the September–December 2024 test set.

Metric	Performance	Interpretation
Accuracy	82.3% ± 2.1%	Exceeded target benchmark of 80% (*p* < 0.05)
ROC-AUC multiclass	0.847 ± 0.023	Excellent discriminative capability (95% CI: 0.824–0.870)
Macro Precision	0.789 ± 0.034	Consistent performance across all stress classes
Macro Recall	0.813 ± 0.028	Balanced sensitivity to all stress categories

**Table 5:** Statistical significance of model comparisons.

Model Comparison (Cohen’s k)	Δ AUC	95% CI of Difference	*p*-Value	Effect Size
LSTM + FFT vs. RF + FFT	+0.057	[+0.032, +0.082]	0.003	0.72 (medium–large)
LSTM + FFT vs. CNN + FFT	+0.077	[+0.051, +0.103]	<0.001	0.89 (large)
LSTM + FFT vs. XGBoost + FFT	+0.067	[+0.041, +0.093]	<0.001	0.81 (large)
LSTM + FFT vs. LSTM	+0.106	[+0.078, +0.134]	<0.001	1.23 (very large)

**Note:** *p*-values adjusted with Holm–Bonferroni for m = 4 pairwise comparisons (LSTM + FFT vs. RF + FFT, CNN + FFT, XGBoost + FFT, and LSTM).

**Table 6:** Per-class precision, recall, and F1 with 95% CIs on the September–December 2024 test set for normal, mild, and high stress levels.

Stress Level	Precision (95% CI)	Recall (95% CI)	F1-Score (95% CI)	Clinical Interpretation
Normal	0.89 (0.86–0.92)	0.94 (0.91–0.96)	0.91 (0.89–0.93)	Excellent detection of baseline states
Mild	0.78 (0.74–0.81)	0.73 (0.69–0.77)	0.75 (0.72–0.78)	Moderate performance reflects transitional nature
High	0.71 (0.67–0.75)	0.77 (0.73–0.81)	0.74 (0.71–0.77)	Balanced sensitivity/specificity for critical cases

**Table 7:** Technical specifications.

Metric	Performance	Significance
One hour prediction	78.6%	Enables proactive management
Computational latency	<2 s	Suitable for edge deployment
Prediction consistency	89%	Robust to temporal variability

**Table 8:** Cross-validation of stress classifications.

Validation Method	Normal (n = 4932)	Mild (n = 1438)	High (n = 480)	Cohen’s κ	*p*-Value
Milk Yield Correlation	89.1%	78.4%	85.2%	0.74	<0.001
Veterinary Assessment	91.7%	82.1%	88.9%	0.79	<0.001
Feed Intake Pattern	88.3%	75.6%	83.1%	0.71	<0.001
Combined Agreement	90.8%	80.9%	87.2%	0.81	<0.001

Note: Agreement percentages represent concordance between circadian-derived classifications and validation methods.

**Table 9:** Ablation study: baselines vs. LSTM + FFT with circadian features.

Model	Accuracy	F1-Macro	ROC-AUC (OvR. Macro)	ΔF1 vs. Proposed	ΔAUC vs. Proposed
Random Forest	0.772	0.742	0.729	−0.058	−0.118
KNN	0.698	0.651	0.517	−0.149	−0.33
CNN	0.699	0.651	0.6	−0.149	−0.247
SVM	0.665	0.641	0.605	−0.159	−0.242
XGBoost	0.681	0.646	0.625	−0.154	−0.222
LSTM	0.799	0.751	0.741	−0.049	−0.106
ANN	0.698	0.651	0.636	−0.149	−0.211
DNN	0.699	0.651	0.732	−0.149	−0.115
Logistic Regression	0.65	NR	NR	NR	NR
Random Forest +FFT	0.78	0.75	0.79	−0.05	−0.057
KNN +FFT	0.72	0.69	0.63	−0.11	−0.217
CNN +FFT	0.76	0.73	0.77	−0.07	−0.077
SVM +FFT	0.7	0.68	0.71	−0.12	−0.137
XGBoost +FFT	0.77	0.74	0.78	−0.06	−0.067
ANN +FFT	0.75	0.72	0.76	−0.08	−0.087
DNN +FFT	0.72	0.672	0.753	−0.128	−0.094
Logistic Regression +FFT	0.535	0.62	0.67	−0.18	−0.177
LSTM + FFT (proposed)	0.823	0.8	0.847	—	—

Note: All algorithms were tested using a circadian (24 h) profile and timed activities.

**Table 10:** Integrated model improvement (covariates).

Model Configuration	Accuracy (%)	Macro-F1	ROC-AUC (OvR)	Δ Accuracy vs. Baseline	Δ F1 vs. Baseline	Δ AUC vs. Baseline	*p*-Value (vs. Baseline)
Baseline (LSTM + FFT only)	82.3	0.8	0.847	—	—	—	—
Extended (LSTM + FFT + Vet Score)	85.1	0.832	0.872	+2.8%	+0.032	+0.025	0.008
Extended (LSTM + FFT + Milk Yield)	84.7	0.828	0.869	+2.4%	+0.028	+0.022	0.012
Optimal (LSTM + FFT + Vet Score + Milk Yield)	87.6	0.861	0.901	+5.3%	+0.061	+0.054	<0.001

**Table 11:** Ablation study on veterinary observations (Vet) and Temperature–Humidity Index (THI) for stress classification: performance metrics across different feature configurations.

Config	Uses Vet	Uses THI	F1-Macro	ΔF1 vs. Full	ROC-AUC	PR-AUC	Accuracy
Full	✓	✓	0.74	—	0.85	0.82	0.823
No Vet	✗	✓	0.71	−0.03	0.83	0.79	0.811
No THI	✓	✗	0.72	−0.02	0.84	0.80	0.816
No Vet and No THI	✗	✗	0.69	−0.05	0.81	0.76	0.802

**Table 12:** Ablation on FFT/circadian features.

Config	A_1_/φ_1_ (24 h)	Harmonics (A_2_,R_12_)	Coherence	F1-Macro	ΔF1	ROC-AUC	PR-AUC
Full	✓	✓	✓	0.74	—	0.85	0.82
No harmonics	✓	✗	✓	0.72	−0.02	0.84	0.80
No coherence	✓	✓	✗	0.72	−0.02	0.84	0.80
No circadian filter	✗	✗	✗	0.68	−0.06	0.80	

## Data Availability

The original contributions presented in this study are included in the article. Further inquiries can be directed to the corresponding authors.

## Abbreviations

The following abbreviations are used in this manuscript.

Acc.	Accuracy
ANN	Artificial Neural Network
CNN	Convolutional Neural Network
DNN	Deep Neural Network
F1-macro	Macro-averaged F1 Score
F1-weighted	Weighted F1 Score
FFT	Fast Fourier Transform
HRV	Heart Rate Variability
IC95%	95% Confidence Interval
KNN	K-Nearest Neighbors
LSTM	Long Short-Term Memory
Macro-Prec	Macro-averaged Precision
Macro Recall	Macro-averaged Recall
OvR	One-versus-Rest
PLF	Precision Livestock Farming
PR-AUC	Precision–Recall–Area Under Curve
RF	Random Forest
RNN	Recurrent Neural Network
ROC-AUC	Receiver Operating Characteristic–Area Under Curve
SVM	Support Vector Machine
THI	Temperature–Humidity Index
XGBoost	eXtreme Gradient Boosting

## Appendix A

**Table A1:** Summary of previous studies on stress detection in dairy cattle using machine learning.

Author/Year	Focus	Method/Algorithm	Stressor or Condition Assessed	Limitations	Key Contribution
Gorczyca et al. (2019) [3,7]	Environmental Stress	ML (Multiple Algorithms)	Heat Stress	Noisy data	Robust model of circadian rhythms + prediction
Wagner et al. (2020) [12]	Circadian Rhythm and Disease	Time-Series Analysis	Stress and Pathologies	Descriptive identification, not predictive	Use of FFT for circadian feature extraction with automated classification
Becker et al. (2021) [17]	Heat Stress Prediction	Random Forest	Heat Stress	Reactive detection, lacks circadian analysis	Integrates circadian rhythms for proactive prediction
Hosseininoorbin et al. (2021) [16]	Behavior Classification	Deep Learning + Time–Frequency Data	General Behavior	Not focused on stress	Integrates general behavior for stress level classification
Brouwers et al. (2023) [14]	Atypical Behavior	Accelerometers + Machine Learning	Postures and Movements	High accuracy but limited to specific behaviors	Generalizes activity (eating, rumination, resting) to model stress
Chapman et al. (2023) [15]	Heat Stress Prediction	Deep Learning	Heat Stress	Point-in-time prediction, lacks circadian context	Prediction of physiological stress with circadian dynamics

## Appendix B

**Formalization of Equations**

Equation (1)—Activity index (PCA-derived weights):

A_t_ = ∑_i_ w_i_ x_i_[*t*], where x_i_[*t*] are hourly durations (eating, resting, rumination, other), and w_i_ are the PCA loadings computed on the first 14 days.

Equation (3)—Discrete Fourier Transform (N = 36, hourly sampling):

$$S[k]=\sum_{t=0}^{N-1}s[t]\cdot e^{-i\;2\;\pi \;k\;t/N}$$

Equation (4)—Dominant circadian peak: *n** = arg max*_n_* |*S*[*n*]|, which should lie near the 24 h rhythm (f_c_ ≈ 1/24 h^−1^ ≈ 0.0417 h^−1^).

Equation (5)—Circadian reconstruction (IFFT with bandpass B[n]):

$s_{circ}\left[t\right]=IFFT\left(B\left[n\right].S\left[n\right]\right),\;whit\;B\left[n\right]=1\;if\;f_{n}\in \;[fmin,fmax]\;$(narrow band around 1/24 h^−1^), and B[n] = 0 otherwise.

Equation (6)—Distance and thresholds between consecutive circadian profiles (z-scored):

$d_{t}\;=\frac{1}{\surd n}\;∥A_{t}-B_{t}\;{∥}_{2}$; thresholds per cow: T_1_ = $\bar{x}$ + s; T_2_ = $\bar{x}$ + 2s.

## Appendix C

**Table A2:** Class-specific precision–recall AUC and additional discrimination metrics.

Stress Level	PR-AUC	95% CI	Specificity	95% CI	NPV	95% CI
Normal	0.943	[0.91–0.97]	0.876	[0.84–0.91]	0.912	[0.89–0.93]
Mild	0.782	[0.74–0.82]	0.891	[0.86–0.92]	0.897	[0.87–0.92]
High	0.728	[0.68–0.78]	0.945	[0.92–0.97]	0.951	[0.93–0.97]
Macro Avg	0.818	[0.78–0.86]	0.904	[0.88–0.93]	0.920	[0.90–0.94]

Note: PR-AUC = precision–recall–area under curve; NPV = negative predictive value. Class imbalance: normal 72%, mild 21%, high 7%.

**Table A3:** Class-specific behavioral profiles (normal, mild, high) summarizing activity peaks, rest periods, rumination reduction, and circadian strength.

Behavioral Metric	Normal Stress Patterns	Mild Stress Patterns	High Stress Patterns
Activity Peaks	Consistent 06:00–08:00 and 16:00–18:00	Phase-shifted (±1–2 h)	High circadian disruption (>3 h shift)
Rest Periods	Stable 22:00–05:00	Increased nocturnal activity (22:00–02:00)	Hyperactivity during typical rest times
Rumination Behavior	Uniformly distributed	15–25% reduction	>40% reduction
Circadian Strength	Robust (*p* < 0.001)	Moderately impaired (*p* = 0.013)	Severely impaired (*p* < 0.001)

**Table A4:** Temporal robustness of the proposed model: seasonal consistency, monthly stability, and error structure.

Seasonal Consistency	81.7% Accuracy	ΔTHI > 8 (Climate Transition)	Maintained Across Temperature Shifts
Monthly Stability	SD < 3%	F = 1.32, *p* = 0.251	Max deviation: 2.8% (Nov)
False Positives	12%	OR = 1.54 [1.12–2.11]	Primarily during dietary changes
False Negatives	8%	HR = 0.87 [0.76–0.99]	Rapid physiological adaptations
Class Confusion	Mild ↔ High: 14%	χ^2^ = 9.41, *p* = 0.002	Normal ↔ Mild: 6%

**Table A5:** Individual cow performance analysis.

Cow ID	Accuracy (%)	AUC	Group Mean Deviation	Percentile Rank	Dunn Post Hoc
4003-Elyn	89.2	0.912	+7.1%	95th	High (Z = 2.34, *p* = 0.019)
479-Mishu	82.1	0.845	±0%	50th	Intermediate (Z = 0.91, *p* = 0.363)
4173-Cris	76.8	0.798	−5.3%	25th	Low (Z = −1.87, *p* = 0.061)

Note: Kruskal–Wallis (one-way, rank) assessed overall differences between groups (H statistic, df = k − 1). When the contrast was significant (α = 0.05), we performed post hoc pairwise Dunn’s test; the z statistic and adjusted *p* (Holm) are reported in the column.

**Table A6:** **Day–night behavioral metrics**.

Metric	Daytime Mean	Daytime 95% CI	Nighttime Mean	Nighttime 95% CI	Δ (Day–Night)	Test	*p*-Value	Effect Size
Circadian amplitude (a.u.)	0.73	[0.68–0.78]	0.62	[0.57–0.67]	+0.11	*t*-test paired	<0.001	0.85
Phase (h)	04:30:00	[04:15–04:45]	16:45:00	[16:30–17:00]	−12.25 h	Circular statistics	<0.001	1.2
Circadian coherence (%)	0.85	[83–87%]	0.78	[75–81%]	0.07	Mann–Whitney U	<0.001	0.92
A–B distance (12 h)	0.42	[0.38–0.46]	0.42	[0.38–0.46]	0.00	Wilcoxon signed-rank	0.89	0.08

**Table A7:** Model performance by period.

Metric	Daytime Mean	Daytime 95% CI	Nighttime Mean	Nighttime 95% CI	Δ (Day–Night)	*p*-Value	Effect Size
F1-Normal	0.91	[0.89–0.93]	0.89	[0.86–0.92]	+0.02	0.032	0.41
F1-Mild	0.75	[0.72–0.78]	0.73	[0.70–0.76]	+0.02	0.12	0.28
F1-High	0.74	[0.71–0.77]	0.77	[0.73–0.81]	−0.03	0.045	0.35
Macro-F1	0.80	[0.78–0.82]	0.79	[0.77–0.81]	+0.01	0.08	0.22
Overall accuracy (micro, 24 h) (%)	82.3%	[80.5–84.1%]	81.7%	[79.9–83.5%]	+0.6%	0.021	0.38
Veterinary Anomaly Score	0.38	[0.32–0.44]	0.52	[0.46–0.58]	−0.14	0.003	0.65
Daily Milk Yield (L/cow)	28.5	[27.1–29.9]	27.2	[25.8–28.6]	+1.3 L	0.018	0.47
Milk Yield Decline vs. Baseline (%)	−1.1%	[−1.8–−0.4%]	−3.2%	[−4.1–−2.3%]	+2.1%	<0.001	0.81
Rumination Time (min/day)	412	[398–426]	389	[375–403]	+23 min	<0.001	0.9

Note: All metrics were evaluated with paired *t*-test.

**Table A8:** Bi-seasonal stratification of stress events and model performance (Ecuadorian Andean climate).

Season	Period	Precipitation	Temperature Range	High Stress Events (n)	Top Triggers	Accuracy	F1-High	AUC
Rainy	January–May, October–December	High (100–150 mm/month)	8–18 °C	327 (68%)	Vaccination (28%), Diet (22%), Health (18%)	82.3%	0.74	0.847
Dry	June–September	Low (20–50 mm/month)	2–20 °C	153 (32%)	Cold stress (35%), Vaccination (23%), Water (15%)	82.6%	0.75	0.849

Note: The equatorial location of the Andes in Ecuador results in two seasons (rainy/dry) instead of four temperate seasons. No significant differences in performance were observed between seasons (Mann–Whitney U = 1.42, *p* = 0.687). Temperature ranges reflect high-altitude Andean conditions at 2950 m.

## Appendix D

Data obtained from smart necklaces during part of the project time.

https://kaggle.com/datasets/5914d76fd65cb7ff8810f4c584e495f47581791521cd805c498eb3316d506f79 (accessed on 17 July 2025)

## Appendix E

The data and interactive visualizations are available in the Carchi State Polytechnic University (UPEC) system through the portal: https://proyectos.upec.edu.ec/ciclos/visualizacion ((accessed on 17 July 2025) (access upon request).
